# COVID-19 and a Window of Opportunity: Guiding Principles for a Health-Promoting Trade Agenda

**DOI:** 10.34172/ijhpm.2020.241

**Published:** 2020-12-05

**Authors:** Joshua S. Yang, Virginia Kotzias, Eric Crosbie, Tim K. Mackey

**Affiliations:** ^1^California State University, Fullerton, Fullerton, CA, USA.; ^2^Norwegian University of Science and Technology, Trondheim, Norway.; ^3^University of Nevada, Reno, Reno, NV, USA.; ^4^Department of Anesthesiology, Division of Infectious Disease and Global Public Health, School of Medicine, University of California, San Diego, San Diego, CA, USA.; ^5^Global Health Policy and Data Institute, San Diego, CA, USA.

 The human and economic toll of coronavirus disease 2019 (COVID-19) has been devastating; as of November 2020, 190 countries have reported nearly 55 million cases of COVID-19 and over one million deaths.^1^ The impacts of COVID-19, however, extend far beyond individual and population-level health, as its disruption to the international trade system has had devastating economic effects^2^; paradoxically, the very rules that govern international trade may pose barriers to states enacting COVID-19 responses that would stabilize international trade. The COVID-19 crisis also highlights how the goals and norms of the international trading system obscure the interdependence between trade and health and can impede states’ abilities to enact structural changes for equitable health and economic systems. This viewpoint demonstrates how COVID-19 has laid bare fundamental problems in the governance of international trade and urges the public health community to use the COVID-19 pandemic as a window of opportunity to advocate for health guiding principles in establishing a more stable, productive, and equitable international trade system.

## COVID-19 and International Trade Policy

 The effects of COVID-19 have been accentuated due to the complex interaction with the international trade system and trade agreements by which the flow of goods, services, and movement of people are regulated. The stress of the pandemic on interconnected and globalized supply chains has already slowed the production of critical supplies and equipment – masks, ventilators, medicines, chemicals – needed to contain the virus and minimize its effects.^3^ Shortages at the beginning of the pandemic sparked export restrictions in 80 countries.^4^ Most have since been repealed, but could be reinstated as the pandemic ebbs and flows, with particularly damaging effect to supply chains in low income countries. Disruptions in global supply chains, travel restrictions, and local lockdown policies have resulted not only in workplace closures and unemployment, but also the loss of billions of dollars in global labor income, increased pressures of the care economy on formal working arrangements, and an estimated 5.2% contraction in global gross domestic product in 2020.^5^ Poor households are hit especially hard by job losses and wage decreases, exacerbating income inequality, and associated health disparities, that may be worsened by international trade.^6^

 As countries move to enact policies to respond to COVID-19, international trade agreements threaten the rights of states to regulate in the public interest. International trade and investment agreements define specific limitations of a state’s right to regulate in order to facilitate trade and investment. Challenges to a state’s sovereign right to regulate based on trade agreements have been made in environmental protection, ensuring access to medicines, and food safety.^7^ For example, Labonté et al have shown how provisions in the recently enacted United States-Mexico-Canada Agreement may limit parties’ ability to regulate sugar sweetened beverages or place prominent warning labels on alcohol containers.^8^ Free trade proponents often point out that existing agreements allow for limits to trade in the interest of public health. But requirements for public health regulations that need to be least disruptive to trade may act as barriers to effectively implementing restrictive public health measures needed in times of health crises such as COVID-19.

 Investor-state dispute settlement (ISDS) is a provision within trade agreements that may impede state action to address COVID-19. ISDS allows commercial entities to directly challenge a nation-state’s public policy in international courts. These efforts have led to blocking, weakening, delaying and most importantly globally preempting the diffusion of best practices to regulate unhealthy commodities both regionally and globally.^9^ During the COVID-19 pandemic, concerns over an imminent wave of ISDS cases has emerged.^10,11^ ISDS cases could arise from governments restricting and closing business activities to limit the spread of the virus and protect workers, securing resources for health systems by requesting the use of private hospital facilities, putting private healthcare providers under public control, or requiring manufacturers to produce ventilators.

 The damage from a COVID-19-related wave of ISDS cases could be significant. Among 1023 ISDS cases, thirteen have resulted in awards or settlements of more than US$1 billion, including for lost future profits.^12^ By the end of 2018, states worldwide had been ordered or agreed to pay investors in publicly known ISDS cases to the amount of US$88 billion. Some developing countries have billions outstanding in pending ISDS claims. This problem is compounded by the increase in third-party funding, comprised of speculative investors who finance ISDS claims and in return receive a percentage of the awarded compensation, incentivizing more ISDS cases.^13^ With government resources stretched to the limit in responding to COVID-19, public money should not be diverted from saving lives and livelihoods into paying ISDS awards or legal fees to fight a claim. In response, a group of 630 organizations from around the world have urged governments to take action to prevent expansion of ISDS during COVID-19, outlining specific recommendations for action (Box 1).

**Box 1. Trade Policy Actions in Response to COVID-19**

**ISDS**^17^

- Permanently restrict the use of ISDS in all its forms in respect of claims that the state considers to concern COVID-19-related measures;
- Suspend all ISDS cases on any issue against any government while it is fighting COVID-19 crises, when capacity needs to be focused on the pandemic response;
- Ensure that no public money is spent paying corporations for ISDS awards during the pandemic;
- Stop negotiating, signing, and or ratifying any new agreements that include ISDS;
- Terminate existing agreements with ISDS, ensuring that ‘survival clauses’ do not allow cases to be brought subsequently.

**IPR**^16,18^

- Waiver of certain TRIPS obligations if related to the prevention, containment, and treatment of COVID-19;
- Exercise of TRIPS flexibilities on a case-by-case or product-by-product basis;
- Invoke TRIPS Article 73 “Security Exception” to override IPRs.

--------------

Abbreviations: COVID-19, coronavirus disease 2019; ISDS, investor-state dispute settlement; IPRs, intellectual property rights; TRIPS, Trade-Related Intellectual Property Rights.

In addition, intellectual property rights (IPRs) provisions in trade agreements, such as the World Trade Organization’s (WTO’s) Trade-Related Intellectual Property Rights Agreement (“TRIPS Agreement”), set minimum standards for member states to harmonize and enforce IPRs, but also include certain public health flexibilities. Together with other clarifications, these “TRIPS flexibilities” allow states to issue compulsory licenses to enable better access to medicines.^14^ However, IPR holders and states that favor more protections have used so-called TRIPS-plus provisions in bilateral and multilateral trade agreements to extend patent protections beyond those required by TRIPS, effectively creating a parallel system of enhanced IPRs utilizing trade negotiation tactics for corporate, and not public health, interests.^14^ With the rapid development and pending approval of COVID-19 therapeutics, diagnostics, and vaccine candidates, debates have already begun about how to ensure equitable access to these potentially life-saving pandemic countermeasures.

 In October 2020, at a TRIPS Council meeting, members discussed how the global IPR system should be used to tackle the COVID-19 pandemic. This included debate regarding a joint proposal submitted by India and South Africa for a temporary waiver of certain TRIPS obligations (including for copyrights, industrial designs, patents, and protection of undisclosed information) that would be applicable for all WTO members if they related to the “prevention, containment or treatment” of COVID-19.^15^ The proposal argues that adaptation of the TRIPS framework is needed to be more responsive to the current pandemic, including ensuring that IPRs do not create barriers to timely and affordable access to COVID-19 health commodities, specifically noting that developing and least developed countries are disproportionately impacted.^16^ Whether the proposal will be adopted, despite opposition from many high-income countries, remains to be seen and will likely be decided later this year or next. Regardless of whether this potential landmark proposal is adopted, other policy options such as TRIPS Article 73 are still available to countries, though they may face practical and legal limitations (Box 1). Hence, the debate about how to ensure equitable access to COVID-19 treatments juxtaposed with whether rights holders will push for added protections is an open and critical question to address as the pandemic moves forward.

## A Health-Promoting Trade Agenda

 The social and economic damage caused by the pandemic will likely continue after the pandemic eventually recedes. Lasting structural effects of the pandemic on global trade will need to be addressed in order to reestablish global and local economies and ensure health benefits of trade are equitably distributed. For global supply chains, this includes interrupted travel and shipping logistics, damage to integrated supply chains, and redirection towards domestic production. Persistent shortages in the global demand of health and other commodities will continue to retract trade for national stockpiling, causing tensions among trade partners, reinforcing existing calls for protectionism and nationalism, and making global cooperation more challenging for other areas of foreign policy and health diplomacy.^19^ Conflict between IPR provisions in trade agreements and national and global procurement mechanisms may slow the spread of necessary health innovation, including those directly aimed to mitigate COVID-19, and monopolies may arise due to overdependence on certain sectors of the economy to the detriment of others.

 A potential opportunity for public health that may result from the COVID-19 pandemic is an increase in the perceived importance of public health to ensuring economic continuity of the international trade system.^20^ With broader acceptance of government intervention to protect against the consequences of COVID-19 and future disease outbreaks, the current political environment may be more responsive to public health concerns to reform trade policy in favor of health security principles, such as pandemic preparedness. For example, access to personal protective equipment, therapeutics, and vaccines are acute challenges for all countries and need to be addressed from human rights, intellectual property, supply chain, and trade perspectives.^21^ Yet, these disparate interests are often incompatible in the current international policy arena, and the international trade system had existing vulnerabilities limiting its effectiveness even prior to COVID-19 that are now attempting to be addressed in the middle of a crisis.

 In the context of the current crisis and global economic slowdown, there seems little chance that renegotiation of the current international trade rules-based system will take place, particularly as the WTO has thus far failed to negotiate a new multilateral trading system since the 2001 Doha Round. The policy actions in Box 1 are clear signs that the current framework of global governance for trade and health is not sufficient. One example of potential international cooperation around these issues is the voluntary COVAX facility initiative to develop and distribute COVID-19 vaccines led by GAVI and the World Health Organization (WHO).^22^ However, even within the context of this new governance mechanism several challenges are emerging, including supporting countries bypassing COVAX to procure vaccines directly with manufacturers and retreating into “vaccine nationalism” in lieu of multilateral cooperation.^23,24^ Additionally, lack of stipulation of IP or pricing restrictions, limited transparency to the terms of advanced purchase agreements, and the hesitancy of the pharmaceutical industry to participate in C-TAP (a COVAX patent pool mechanism aimed at increasing scale-up and access to COVID-19 treatments and vaccines), are emblematic of the challenges faced by these initiatives that straddle global health, trade, and IPR interests.

 Despite these challenges, the public health community should not allow this window of opportunity to reform and optimize global governance to better align shared goals of population health, trade and sustainability, and IPR flexibilities slip away. In fact, a legacy of this pandemic will be how central health does or does not become in the broader functioning of the international economic system; if we ignore global health challenges, it will be to the peril of all nations. Based upon the shortcomings of the international trade system that are now becoming more apparent due to COVID-19, we urge the public health community to engage in political action on trade. Guiding principles for a health-centered trade international trade system with proposals for policy innovation and reform in the context of international trade agreements are listed in Box 2.

**Box 2. Guiding Principles for a Health-Centered International Trade System**

- Implementation of strong and affirmative public health measures should be normalized and explicitly allowable within multilateral, regional, and bilateral trade agreements while also allowing for policy experimentation particularly in the context of responding to health emergencies.
- Public health measures should only be challengeable as a non-tariff barrier to trade if they are shown to have no public benefit, not if they are not least disruptive to trade.
- Trade agreements should apply a public health standard when stipulations for non-tariff barriers to trade are being negotiated, including, but not be limited to:
  ♦ efforts to reduce inequality as a desired outcome of free trade, including redistribution of income, fair compensation, broadening educational and working training opportunities, improving work quality and labor market outcomes, strengthening social safety net systems, and progressive tax and inheritance systems.
  ♦ promotion of worker health protections, including a flexible regulatory infrastructure that promotes worker protections via collective bargaining or other forms of worker representation in the negotiation process, incentives for countries to create stronger legal standards for worker health, or the identification of shared efforts to address social determinants of health that increase occupational risk for disadvantaged groups.
  ♦ incentivization of sustainable production practices, such as use of biodegradable plastics and medical equipment, life cycle energy assessments, environmental targets and sustainability requirements, or other performance thresholds.
- The use of ISDS in all its forms should be permanently restricted in respect of claims that the nation-state considers a health emergency-related measure.
- Future trade agreements should exclude ISDS or at the minimum have a carve out/exclusion for health-related disputes/issues.
- Existing trade agreements with ISDS provisions should be renegotiated to remove ISDS clauses or otherwise terminated, ensuring that ‘survival clauses’ do not allow cases to be brought subsequently.
- TRIPS-flexibilities should either be strengthened and clarified in the context of health emergencies, which should include allowing countries greater ability to quickly issue compulsory licenses on a blanket exception (ie, instead of on a case-by-case basis), possible revision and expanding the scope and expediency of TRIPS paragraph 6 system, and exclusion of TRIPS-plus provisions for health emergency purposes.
- Consideration of instituting an automatic temporary waiver of TRIPS IPR obligations limited in duration and applicable for health commodities in the event of a health crisis that constitutes a Public Health Emergency of International Concern under the WHO International Health Regulations or a “emergency in international relations” under TRIPS Article 73.

--------------

Abbreviations: ISDS, investor-state dispute settlement; IPR, intellectual property rights; TRIPS, Trade-Related Intellectual Property Rights; WHO, World Health Organization.

## Conclusion

 The COVID-19 pandemic has made more apparent how international trade agreements threaten public health. Though ISDS and IPRs have been highlighted, the rights of states to regulate in the public interest extend to other elements of trade policy as well, such as good regulatory practice. A new set of guiding principles for the international trade system will require a fundamental shift in thinking about the purpose of international trade and its role in the current and future “health” of global and national economies. Preventing non-tariff barriers to trade has driven an overreach of efforts to ensure investors’ ability to maximize return on investment, the shortcomings of a dogma of ever freer trade having been exacerbated by COVID-19. What a different model of trade may look like is an unanswered question. Instead, we suggest that the design and enforcement of trade agreements should not only allow but promote state efforts to engage in policy activity that promotes public welfare and population health through a vision of health creation alongside equitable and sustainable economic development. This can only be achieved through a new view of trade agreements, not as a mechanism of achieving primarily economic efficiency, but as instruments of advancing human-centered social and economic policies.

## Ethical issues

 Not applicable.

## Competing interests

 Authors declare that they have no competing interests.

## Authors’ contributions

 JSY, VK, EC, and TM conceptualized and designed the study, and drafted and revised the manuscript. JSY supervised manuscript preparation and finalization.
